# [Expression of Concern] Effect of fibroblast co-culture on the proliferation, viability and drug response of colon cancer cells

**DOI:** 10.3892/ol.2026.15680

**Published:** 2026-06-03

**Authors:** Byumseok Koh, Hyojin Jeon, Dahee Kim, Dukjin Kang, Kwang Rok Kim

Oncol Lett 17: 2409–2417, 2019; DOI: 10.3892/ol.2018.9836

Following the publication of the above paper, it has been drawn to the Editor's attention by a concerned reader that, regarding the morphological images of the SW480 cells shown in Fig. 1A on p. 2411, the ‘Day 4/SW480+BJ’ and ‘Day 4/SW480+CCD-16Co’ data panels were apparently matching, suggesting that this figure had been assembled incorrectly

The authors have been contacted by the Editorial Office to offer an explanation for this apparent anomaly in the presentation of the data in this paper, and we are awaiting their response. Owing to the fact that the Editorial Office has been made aware of this potential issue surrounding the scientific integrity of this paper, we are issuing an Expression of Concern to notify readers of this potential problem while the Editorial Office continues to investigate this matter further.

